# The Seven Deadly Sins of Measuring Brain Structural Connectivity Using Diffusion MRI Streamlines Fibre-Tracking

**DOI:** 10.3390/diagnostics9030115

**Published:** 2019-09-06

**Authors:** Fernando Calamante

**Affiliations:** 1 Sydney Imaging, The University of Sydney, Sydney, New South Wales 2050, Australia; fernando.calamante@sydney.edu.au; Tel.: +61-2-9114-4293; 2 School of Aerospace, Mechanical and Mechatronic Engineering, The University of Sydney, Sydney, New South Wales 2006, Australia; 3 Florey Department of Neuroscience and Mental Health, University of Melbourne, Melbourne, Victoria 3052, Australia; 4 Brain and Mind Centre, The University of Sydney, 94 Mallett Street, Camperdown, NSW 2050, Australia

**Keywords:** fibre-tracking, tractogram, connectivity, tractography, streamlines

## Abstract

There is great interest in the study of brain structural connectivity, as white matter abnormalities have been implicated in many disease states. Diffusion magnetic resonance imaging (MRI) provides a powerful means to characterise structural connectivity non-invasively, by using a fibre-tracking algorithm. The most widely used fibre-tracking strategy is based on the step-wise generation of streamlines. Despite their popularity and widespread use, there are a number of practical considerations that must be taken into account in order to increase the robustness of streamlines tracking results, particularly when these methods are used to study brain structural connectivity, and the connectome. This review article describes what we consider the *‘seven deadly sins’* of mapping structural connections using diffusion MRI streamlines fibre-tracking, with particular emphasis on ‘sins’ that can be practically avoided and they can have an important impact in the results. It is shown that there are important ‘deadly sins’ to be avoided at every step of the pipeline, such as during data acquisition, during data modelling to estimate local fibre architecture, during the fibre-tracking process itself, and during quantification of the tracking results. The recommendations here are intended to inform users on potential important shortcomings of their current tracking protocols, as well as to guide future users on some of the key issues and decisions that must be faced when designing their processing pipelines.

## 1. Introduction

Diffusion MRI encodes the random microscopic motion of water molecules along a particular (diffusion-weighted gradient) direction. In white matter in the brain, this motion is more freely along the axonal fibres (rather than across the fibres), and the diffusion MRI signal intensity thus depends on the orientation of the structures relative to the diffusion gradient direction: higher diffusion (e.g., along the direction of the axons) leads to lower diffusion MRI signal intensity (i.e., more signal attenuation). Diffusion MRI therefore provides an imaging contrast that can be used to investigate tissue microstructural organisation, and in turn investigate structural connectivity in the brain [1].

Diffusion MRI has made a tremendous impact to the way we study structural brain connections in the healthy brain, as well as how these connections are disrupted in the abnormal brain—the reader is referred to [1] for a comprehensive review book on this topic. The pioneering work by Basser and colleagues [2,3] with the introduction of the tensor model to describe the anisotropic nature of the diffusion MRI signal in white matter, in what is now known as Diffusion Tensor Imaging (DTI), opened up the possibility of mapping white matter pathways non-invasively, by using an approach referred to as fibre-tracking.

Arguably the most commonly used strategy for achieving fibre-tracking using diffusion MRI relies on the step-wise generation of streamlines [4]. This approach involves generating a streamline (or track) from a starting location (known as the ‘seed’), and advancing the location step-by-step (with a step length known as the ‘step size’) in a direction computed from the information contained in the diffusion MRI data (e.g., the principal direction of the diffusion tensor, for the case of DTI). This process is continued, usually within some constrains (e.g., a restriction in the maximum curvature allowed between steps, usually referred as a curvature constraint), until a stopping criteria is reached (e.g., the streamline reaches a location with very low diffusion anisotropy, for the case of DTI-based fibre-tracking). 

**Figure 1:**
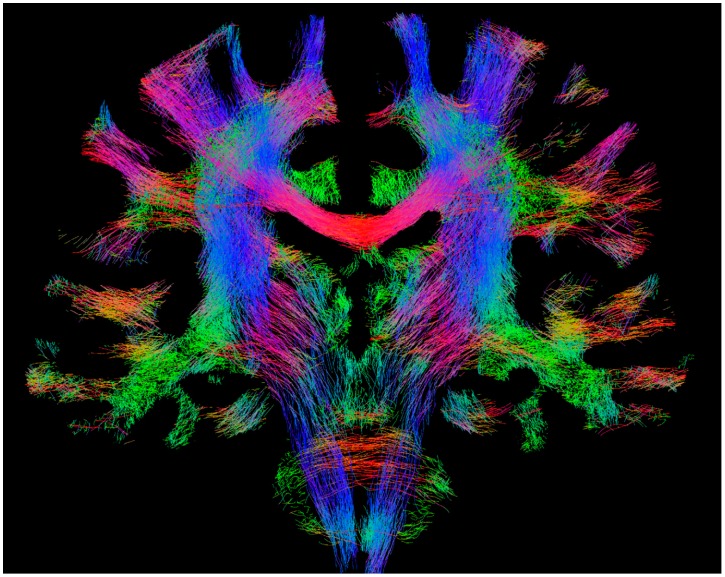
Example whole-brain fibre-tracking results (also referred to as ‘tractogram’) from human healthy subject scanned at 3T. The results shown correspond to 100,000 streamlines from probabilistic tracking, displayed as a 4-mm coronal section. The colour-coding corresponds to the fibre orientation (red: left–right, green: dorsal–ventral, blue: cranial–caudal). Figure previously published in [14], with permission of Springer, 2017.

This type of streamlines fibre-tracking can be broadly divided into deterministic and probabilistic, depending on how the direction to progress at each step is selected [4]. In the deterministic approach [5,6,7], the direction to be chosen at each step is uniquely defined (and thus its ‘deterministic’ name). (In this case, multiple repeats of a streamline generation process starting from a given seed location leads to exactly the same streamline solution.) For example, this is the case for DTI algorithms that solely consider the direction corresponding to the largest eigenvalue of the tensor, regardless of the actual shape of the tensor or the noise in the data. In contrast, in the probabilistic approach [8,9], the direction to be followed at each step is not considered to be uniquely defined, and the diffusion MRI data are instead assumed to provide an estimate of the distribution of possible directions to follow (and thus its ‘probabilistic’ name) (In this case, multiple repeats of a streamline generation process starting from a given seed location will lead to different streamline solutions, reflecting the uncertainty present in the data.). Both approaches have been combined with various models used to determine local fibre orientations. For example, probabilistic approaches have been combined with the information from the tensor model [8,9,10,11], with the orientation distribution function (ODF) from Q-ball imaging [12], and with the fibre orientation distribution (FOD) from the spherical deconvolution approach [13], among others. Figure 1 shows an example image from streamlines fibre-tracking, displaying a slab of the results of whole-brain tracking, which is often referred to as the ‘tractogram’.

While this tracking strategy is very simple in nature, it has been the most widely used approach for over two decades, since the early studies in the late 1990s [5,6,7]. There are however a number of practical considerations that must be considered in order to increase the robustness of the tracking results. These issues are particularly relevant when fibre-tracking results are used to study brain structural connectivity, and the structural connectome (The term connectome is used to refer to a comprehensive description of the structural connections in the brain, usually represented by a connectivity matrix, which can be subsequently analysed using graph theoretical tools [15,16]). 

This article describes, in the author’s opinion, what it could be considered the *‘seven deadly sins’* of mapping structural connections using diffusion MRI. It is intended to inform users on some potential important shortcomings in their current protocols, as well as to guide future users of key issues and decisions that must be faced when starting in this field. It should be noted, however, that this article is not intended to be a comprehensive review of all aspects related to fibre-tracking, e.g., a description of the different type of algorithms (and their pros/cons), all possible parameter choices and their impact (e.g., step size, curvature constrains, number of tracks), the impact on fibre-tracking of all aspects of the acquisition protocol (e.g., field strength, spatial resolution, imaging readout strategy), etc., and the reader is referred to recent review articles and specialist books, where many of these issues are covered in detail [1,4,13,17,18,19]. Similarly, this article does not focus on ‘sins’ that cannot be practically avoided or on intrinsic limitations that affect every tracking algorithm (e.g., the difficulty of distinguishing kissing from crossing fibres scenario), as there is very limited choice the user can have to influence their impact. This article instead focusses on summarising some of the key issues (i.e., ‘sins’) that can be practically avoided/minimised, and includes simple illustrative examples to demonstrate the major impact they can have on the tracking results. 

## 2. Seven Deadly Sins 

We describe in this section what, in the author’s opinion, constitute the main issues to avoid (i.e., the ‘deadly sins’) when performing streamlines fibre-tracking in the context of brain structural connectivity, as their choice can have very practical impact on the results. As described in the previous section, it should be noted however that not every choice that can have an impact on the results will be discussed (e.g., choice of step-size, which can have a major impact on the tracking outcome), as many of these choices are usually now properly selected. We focus here on some of the key issues that, despite being often well-recognised in the technical literature, their inappropriate choice is unfortunately still lingering in many application studies. This article is aimed at drawing attention to such users about aspects of their protocols that they should be seriously considering changing to increase the robustness of their results.

### 2.1. Model to Estimate Local Fibre Directions—Deadly Sin 1

As described in the Introduction section, one of the central steps in any streamlines tractography algorithm is selecting the direction to extend the streamline at each step. Traditionally, this has been based on the primary orientation of the tensor using DTI (i.e., the eigenvector corresponding to the largest eigenvalue) [5,6,7,20]. However, while the tensor model is appropriate for characterising the main orientation of the white matter fibres in areas containing a single fibre population, it has been now extensively demonstrated that it fails to characterise the more complex configurations when multiple fibre populations are present in a voxel (e.g., in the often called crossing fibre regions) [21,22]. This problem reflects the intrinsic limitations of this simple model, as the tensor can only have a single primary orientation, and when more than one fibre population is present, the tensor will appear oblate (e.g., with two-fibre crossing regions) or spherical (e.g., with three-fibre crossing regions), with the primary orientation of the resulting tensor failing to properly identify the main direction of any of the constituent fibre populations. 

As it is often the case, the solution to this problem is to consider a more advanced model, which is able to better characterise these more complex configurations involving multiple fibre populations. A number of such models have been proposed, including multi-tensor models [23], spherical deconvolution [24], Q-ball imaging [25], persistent angular structure MRI [26], and diffusion spectrum imaging [27], among others (There is yet no general consensus as to which of these advanced models has the best performance, and they differ in their data requirements, computational demands, minimal angular resolution they are able to resolve, etc. A discussion of their relative advantages/disadvantages is beyond the scope of this article; however, the key point they share for the purpose of the topic covered here is that they are all designed to model multiple fibre populations within a voxel).

Many of these more advanced models allow to compute the FOD function (also referred to as fibre Orientation Distribution Function, fODF), which, as the name states, it describes the fibre orientations present within the voxel [22]. For example, the spherical deconvolution approach [21,24,28], one of these advanced models that is currently widely used for characterising local fibre orientations, allows to estimate the FOD at each voxel location. Importantly, this can be done without any prior assumption as to the numbers of fibre bundles present in the voxel (i.e., the data themselves determines the number of different fibre populations). The FOD provides information about the number of fibre populations, their relative proportions and directions, as well as a measure of the uncertainty in the direction estimates (e.g., the wider the FOD, the larger the uncertainty) (Recent variants of the spherical deconvolution approach even allow to account for the partial volume contribution within the voxel from an isotropic component (e.g. grey matter and cerebrospinal fluid) [29]).

These advanced models are therefore able to overcome a major limitation intrinsic to the tensor model, by being able to characterise multiple fibre populations. Despite the benefits of these more advanced models, and that they have now been implemented in many freely available software tools (examples of freeware tools include: Dipy (http://nipy.org/dipy/), MRtrix (http://www.mrtrix.org/), ExploreDTI (http://www.exploredti.com/), FDT (https://fsl.fmrib.ox.ac.uk/fsl/fslwiki/FDT), Camino (http://camino.cs.ucl.ac.uk/), and TrackVis (http://trackvis.org/). Note, however, that this is not meant to represent an exhaustive list, but just example tools.), it is still not uncommon to encounter fibre-tracking studies that are based on the tensor model. Given the above arguments, we believe the tensor model should be considered an unsuitable model when mapping structural connections in the brain using streamlines fibre-tracking [12,30,31,32,33,34], and it is therefore the *first ‘sin’* in the list. 

It should be emphasised, however, that this does *not* mean that the use of DTI more broadly should be considered a sin. For example, DTI provides useful metrics that can still play a very important role in many other applications, such as is the case for the trace of the diffusion tensor, which is a widely used parameter in the management of acute stroke imaging [35]. Similarly, we do not imply that DTI may not be suited for tracking in other organs with different fibre architecture complexity. It is the use of DTI in the context of streamlines fibre-tracking in the brain the one that is being criticised here.

**Figure 2:**
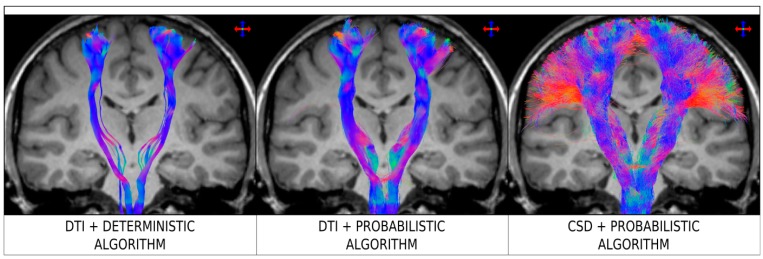
Fibre-tracking results on a healthy human subject obtained using a seed region in the brainstem and target region in the sensorimotor cortices. Coronal T1-weighted images overlaid with tractography results for Diffusion Tensor Imaging (DTI) combined with a deterministic algorithm (left), DTI combined with a probabilistic algorithm (middle), and constrained spherical deconvolution (CSD) combined with a probabilistic algorithm (right). Data were acquired using 60 diffusion-weighted directions with *b* = 3000 sec/mm^2^. The colour-coding indicated the fibre orientation (same convention as in Figure 1). Figure modified from a figure previously published in [30], with permission of American Association of Neurological Surgeons, 2013.

Figure 2 illustrates the practical consequences of using the traditional (and, unfortunately, still often used) DTI streamline tracking approaches, based on the tensor model (both combined with deterministic and probabilistic tracking algorithms). The example corresponds to tracking from the brainstem to the sensorimotor cortices. As can be seen in the figure, the DTI tracking results are only able to reconstruct the medial part of the corticospinal tract, but fail to reconstruct the lateral projections towards areas such as those involved in fingers, brow, eye, face, lips, jaw, tongue, and swallowing. The reason for this is because the tensor model is unable to characterise the complex fibre architecture in areas of the centrum semiovale, where the corpus callosum, cortico-spinal tract and superior longitudinal fasciculus cross paths. (The specific acquisition parameters (e.g. field strength, number of directions, *b*-value, etc.) and tracking parameters (e.g. step-size, number of streamlines, termination criteria, etc.) used in Figure 2 are not explicitly listed here (although they can be found in the original source article for that figure), as the general conclusion from the figure is not greatly influenced by this specific choice, but rather the figure illustrates a general behavior for this tracking scenario. In particular, failure to reconstruct lateral projections of the corticospinal tract is a limitation of DTI deterministic streamlines tracking [30,31,36,37], regardless of the specific implementation or choice of parameters). Importantly, while the uncertainty introduced by a probabilistic tracking algorithm could be expected to partly address the intrinsic limitation of DTI in multi-fibre regions, the results from Figure 2 clearly illustrate that the use of probabilistic tracking in itself does not overcome the limitations of the tensor model (e.g., the lateral projections of the cortico-spinal tract are still not recovered by combining DTI and probabilistic tracking, a result consistent with other studies [31,32,33,37]). The use of a higher order model is thus warranted (e.g., right panel in Figure 2).

**Figure 3:**
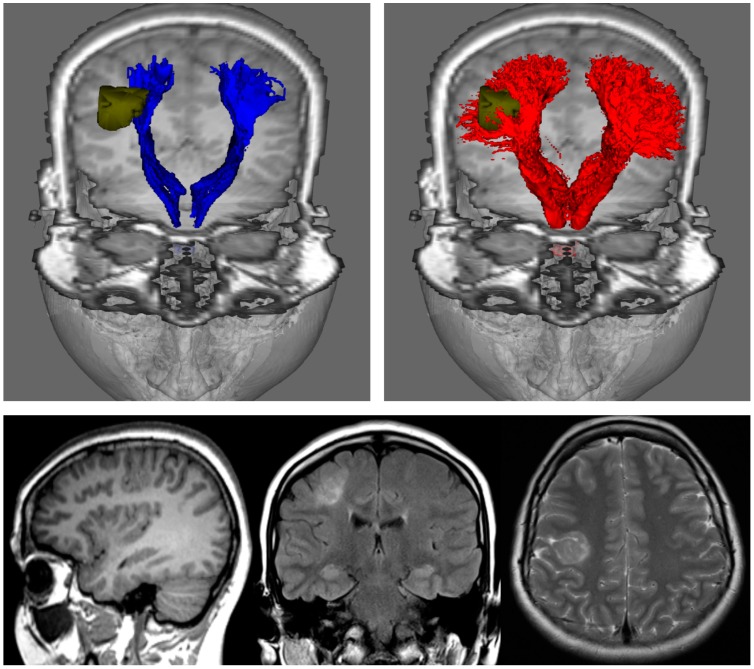
Fibre-tracking results on a 24-year-old woman with a right focal cortical dysplasia situated in the right posterior frontal lobe (see images in bottom row). These results were obtained using a seed region in the brainstem and target regions in the sensorimotor cortices. DTI-based tractography (blue) and CSD-based tractography (red) were derived from the same diffusion MRI data set); segmented pathology volumes (green) overlaid on T1-weighted image. DTI-based tractography suggests that only the medial aspect of the lesion impinges on the corticospinal tracts, whereas CSD-based tractography suggests that the lesion is enveloped by medial and lateral projections. DTI: Diffusion Tensor Imaging; CSD: constrained spherical deconvolution. Note that while only DTI combined with deterministic tracking is shown in the top-left image, the results of combining DTI with probabilistic tracking are expected to be very similar (cf. left and middle images in Figure 2). Figure modified from a figure previously published in [30], with permission of American Association of Neurological Surgeons, 2013.

The results from Figure 2 highlight two important practical consequences of using the tensor model for tracking. First, the estimated connectivity will fail to properly represent connections (and, importantly, this is often the case for major connections) present in the brain and, thus have serious consequences, for example, for studies focusing on the connectome (as these connections will be missing from the connectivity matrix). Secondly, from a clinical point of view, these missed connections can also have very serious consequences, as illustrated by Farquharson et al. [30] with diffusion MRI data from patients undergoing pre-surgical imaging assessment (see Figure 3): while the results from DTI-based fibre-tracking suggest that there is a safe margin of resection in the example shown from an epilepsy patient with focal cortical dysplasia, the analysis of the same diffusion MRI data using spherical deconvolution suggests that the lesion is surrounded by the projections of the corticospinal tract. These general findings are consistent with those from other related studies [31,37].

It should be emphasised however that we are not implying that these more advanced models (e.g., those that compute the FOD) are not error proof, but rather that they are in principle able to account for multiple fibre populations, a feature the tensor model is not able to achieve, thus addressing one of the important shortcomings of the tensor model in the fibre-tracking context. 

The tensor model should therefore be avoided when using diffusion MRI for mapping brain connectivity using streamlines fibre-tracking (It is worth emphasising that the term DTI is often misused in this context. DTI should only refer to the use of diffusion MRI when the data are modelled using the tensor. Therefore, it is inappropriate to refer to DTI in the more general scenario when non-tensor models are used, and the wider term ‘diffusion MRI’ should be used instead).

An interesting issue relates to the prevalence in the brain of the tensor problem in fibre-tracking. It had been often assumed that the limitations of the tensor model did not have a major practical impact on most fibre-tracking results. To test the validity of this assumption, a number of studies focussed on characterising the prevalence of the problem, such as by calculating the proportion of white matter voxels that contains more than one fibre population, for the typical spatial resolution used in human brain diffusion MRI studies. In particular, Jeurissen et al. [38] nicely demonstrated that up to 90% of the white matter voxels can contain more than one fibre population. This implies that it is arguably almost impossible to imagine a white matter bundle that will not require to traverse voxels containing a different fibre population at some point during its length, and thus traverse a region where the tensor model is inappropriate. In fact, it is most often the case that major white matter bundles will occupy numerous voxels that contain multiple fibre populations, and therefore voxels where the tensor model in unsuitable to properly characterise the underlying local fibre architecture.

While the actual proportion of white matter voxels containing more than one fibre population is certainly dependent on the spatial resolution of the data (i.e., the higher the resolution, the smaller the percentage of affected voxels), the prevalence of the tensor problem cannot be eliminated solely by using higher spatial resolution, given that, for example, there are brain regions where white matter interdigitation takes place: regions with multiple fibre populations will be therefore always present for the spatial resolution practically achievable with diffusion MRI. 

Neglecting the impact of the problem associated with the intrinsic limitations of the tensor model described in sin 1 by underestimating its prevalence within the brain can therefore have serious consequences. 

### 2.2. Consistent Results Do Not Mean Accurate Results—Deadly Sin 2

Importantly, many of the errors associated with the tensor model, and particularly when combined with deterministic streamlines, which is the most commonly used case for DTI tracking, lead to *biased* results. For example, the failure of DTI tracking to identify the lateral projections of the corticospinal tract described in the previous section is a *systematic* effect: as shown by Farquharson et al. [30], DTI tracking consistently failed to reconstruct the lateral projections in every single subject studied, as this relates to an intrinsic limitation of the tensor model to accurately model the complex fibre architecture in the centrum semiovale region; this finding was consistent with other reports [31,37]. Importantly, this ‘consistency’ was previously used as evidence in support of the good qualities of fibre-tracking results [39]. However, it should be emphasised that consistent tracking results do not imply reliable tracking; this relates to the case of trading bias for precision: DTI tracking can lead to very consistent and reproducible cortico-spinal tract reconstructions, but always missing the lateral projections (i.e., always the same answer, but the wrong answer).

While very high reproducibility is a very important feature of any tracking algorithm [40], its reliance as key quality measure (particularly, when this is at the expense of other important qualities, such as accuracy) when studying connectivity is considered here the *second ‘sin’* in the list.

One possible way to assess the accuracy in which different tractogram reconstructions (e.g., from different tracking algorithms or different local fibre orientation models) are supported by the underlying diffusion MRI is using the LInear Fascicle Evaluation (LiFE) method [41]. This method uses a forward model to predict the diffusion MRI signal that would be measured from a set of streamlines. The LiFE method shares similarities with other related approaches [42,43], in that they attempt to assign appropriate weights to a set of streamlines to match the associated signal computed from the resulting tractogram to the actual signal of the acquired diffusion MRI data (see deadly sin 6 below for further details). 

### 2.3. Acquisition Protocol for Fibre-Tracking Applications—Deadly Sin 3

**Figure 4:**
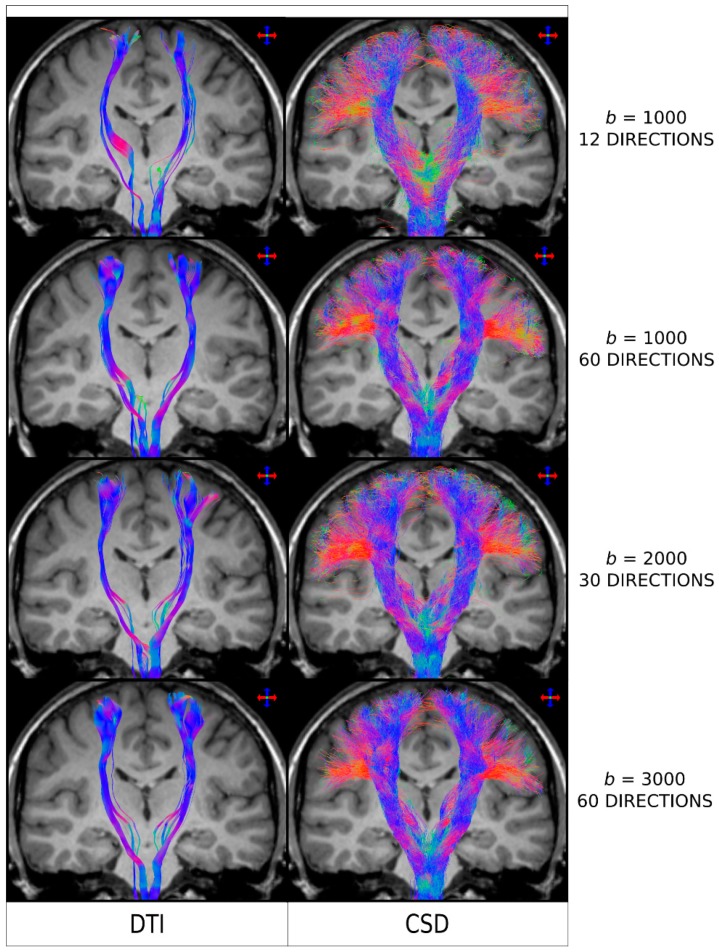
Fibre-tracking results on a healthy human subject obtained using a seed region in the brainstem and target regions in the sensorimotor cortices. Coronal T1-weighted images overlaid with tractography results using DTI combined with a deterministic algorithm (left column) and CSD combined with a probabilistic algorithm (right column), for a range of diffusion MRI protocols (as indicated by the b-value, in s/mm^2^, and number of directions). The colour-coding indicates the fibre orientation (same convention as in Figure 2). DTI: Diffusion Tensor Imaging; CSD: constrained spherical deconvolution. As clarified for the interpretation of the results from Figure 3, and based on the results reported in Figure 2, the general conclusions from this figure are also expected to be applicable to the case of combining DTI with probabilistic tracking. Figure modified from a figure previously published in [30], with permission of American Association of Neurological Surgeons, 2013.

As it is the case with other MRI analysis methods, the better the acquired diffusion MRI data, the better the fibre-tracking results will be. When it comes to diffusion MRI data, two specific parameters have a major impact on the outcome results: the b-value (which determines the degree of diffusion weighted sensitisation), and the number of diffusion-weighted encoding directions. Both parameters have been shown, for example, to influence the achievable angular resolution of the resulting FOD [28]. In particular, if the b-value is too low and/or the number of directions too few (such as it was the case for early DTI protocols, e.g., with 10–20 directions a *b*-value < 1000 s/mm^2^), then the reconstructed FODs will be very broad [21,28], in turn leading to greater spread of the fibre-tracking results, lower power to resolve crossing fibres, and noisier streamlines when using a probabilistic fibre-tracking approach [13,30]. This is illustrated in Figure 4, where it can be seen that the choice of b-value and number of directions has a major impact in the quality of the tracking results Interestingly, the tracking results from Figure 4 (and the results shown in more detail in reference [30]), demonstrated that even for data acquired from protocols that could be considered highly unsuitable for higher-order models (e.g. only 12 directions and *b* = 1000 s/mm^2^—top row in Figure 4) and, thus, expected to highly favour DTI-based analysis, CSD-based results were still superior to DTI-based results: CSD-based analysis was able to reconstruct lateral projections successfully (albeit more noisier than with a HARDI type acquisition), while these were not reconstructed using DTI.

The optimal acquisition protocol can depend on the model used to analyse the data. For example, for the commonly used spherical deconvolution approach, Tournier et al. [44] demonstrated that b-value ~3000 s/mm^2^ provides the optimal angular resolution for typical acquisition protocols in clinical scanners; for this b-value, it was also shown that a minimum of 45 gradient directions is required. So, while the analysis of retrospective data might need to accept compromises in data quality (e.g., data were acquired before the requirements for the more advanced models have been characterised), it is important that protocols for prospective data acquisition are now carefully tailored to the intended analysis. If structural connectivity based on streamlines tracking is the ultimate aim, a protocol with high angular resolution (often referred to as High Angular Resolution Diffusion Imaging, or HARDI) should be employed, and the b-value must be optimally chosen for the advanced model to be used (e.g., *b*-value ~3000 s/mm^2^ for spherical deconvolution analysis). Otherwise, the resulting sub-optimal data quality will have a major impact on the reliability of the tracking results. 

It should be noted, however, that the optimal protocol can be different for different multi-fibre models. For example, a denser sampling with higher maximum *b*-value has been recommended for diffusion spectrum imaging [45], and a multi-shell HARDI acquisition (i.e., acquisition of multiple directions at more than one non-zero *b*-value) has been shown to be required for robust reconstruction of multi-tensor approaches [46]. 

Given the major consequences the acquisition protocol can have in fibre-tracking outcomes, selection of a sub-optimal diffusion MRI protocol is therefore considered the third ‘sin’ in the list.

### 2.4. Criteria for Streamline Termination—Deadly Sin 4

**Figure 5:**
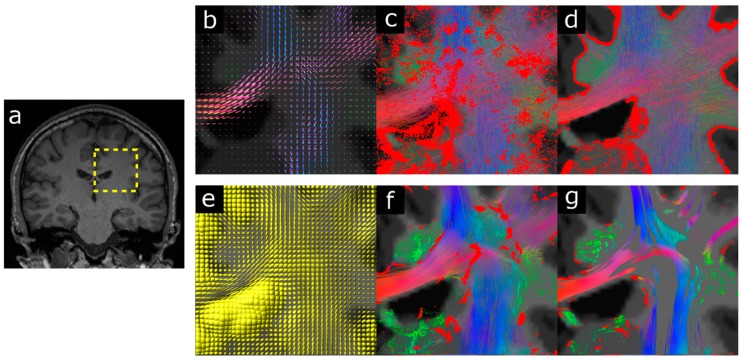
Demonstration of the effects of incorporating anatomical priors during fibre-tracking. (**a**) Coronal T1-weighted anatomical image highlighting by the yellow dotted lines the zoomed region. (**b**) Fibre Orientation Distributions on the zoomed region, overlaid on the segmented anatomical image. (**c**) Streamlines computed using probabilistic tracking without incorporating anatomical priors. (**d**) Corresponding streamlines from probabilistic tracking when the Anatomically Constrained Tractography (ACT) framework is considered. (**e**) Diffusion tensors on zoomed region. (**f**) DTI-based deterministic fibre-tracking results without incorporating anatomical priors. (**g**) Corresponding DTI-based streamline results when the ACT framework is considered. Streamline terminations are indicated by the red dots. For all cases, only streamlines within a 0.9 coronal slice are shown. Figure modified from a figure previously published in [47], with permission of Elsevier Inc, 2012.

A well-recognised problem when using fibre-tracking results to investigate structural connectivity is that streamlines often can have ill-defined end-points (e.g., they can terminate in the middle of white matter or penetrate into the cerebrospinal fluid spaces in the ventricles) or be found to cross sulci, both of which are non-physical scenarios for real axonal pathways. One way to overcome these limitations is to incorporate anatomical information (e.g., from high-resolution T1-weighted images) to inform the type of streamline propagation that is allowed during fibre-tracking. This is the approach that has been adopted by a number of methods, such as Anatomically Constrained Tractography (ACT) [47], and Continuous Map Criterion (CMC) [48], among others [49,50]. In this way, well-defined criteria for streamline termination can be incorporated *during* the tracking process, and thus generate a streamline set that, for example, terminates at the grey/white matter interface or in sub-cortical grey matter (with no streamline terminating in the middle of white matter or cerebrospinal fluid spaces), as well as eliminating the possibility of streamlines unrealistically crossing sulci (see Figure 5, top row). These type of fibre-tracking strategies that incorporate anatomical priors in the tracking process thus lead to much more truthful representations of white matter pathways, as these are made self-consistent with other available anatomical information. 

As the absence of this extra anatomical information as part of the termination criteria during the tracking process can lead to a number of physically unrealistic pathway reconstructions and termination points [47,48], it is therefore considered the forth ‘sin’ in the list.

Another interesting insight gained from the behaviour of tracking algorithms such as ACT and CMC relates to the proportion of streamlines suitably assigned to connectivity matrices when studying the connectome. Ideally, each streamline in the tractogram should be assigned to two (and only two) nodes in the connectome: if it is assigned to fewer nodes, it means that one of its end points did not terminate within a parcellation node; if it is assigned to more than two nodes, it implies that the streamline crossed multiple parcellation nodes during its transit, i.e., implying it had multiple synapses. In a recent study, Yeh et al. [51] showed that when ACT was not used (and even if advanced methods including spherical deconvolution and probabilistic tracking were considered), the proportion of streamlines connecting two nodes was between ~25–65% (depending on whether the FreeSurfer or Automated Anatomical Labeling parcellations were used); the proportion increased to >90% when ACT was included. These results once again highlight that, when sub-optimal tracking methods are used, very large portions of the reconstructed tractogram (and therefore of the underlying white matter pathways) will not be reliable represented in the connectome. This stresses the importance of avoiding deadly sin number four.

It should be emphasised that the problems associated with using a poor termination criteria affect most step-wise streamline tracking algorithms, including those based on using advanced models to analyse HARDI data combined with probabilistic fibre-tracking (e.g., see erroneous streamline terminations in Figure 5c). They need to be carefully considered regardless of the choice of model to estimate fibre orientations during the tracking step.

Interestingly, the impact of incorporating anatomical information during the tracking process can also provide useful insights regarding the limitations and problems associated with standard DTI-based fibre-tracking. For example, Smith et al. [47] demonstrated that the combination of ACT with deterministic DTI fibre-tracking leads to the removal of streamlines from large sections of the whole-brain tractogram (see Figure 5, bottom row); while this may initially seem to suggest that ACT is not beneficial for this type of tractography algorithms, it is in fact highlighting the reality that large sections of white matter cannot be reliably reconstructed using standard DTI-based fibre-tracking, as the streamlines generated in those sections will never terminate in meaningful areas or, alternatively, will traverse unrealistic regions at some point of its path. It is important to recognise that, to some extent, this will be always the case for results from any deterministic streamlines algorithm, as there will always be sections of white matter which contain streamlines that end (or traverse through) physically unrealistic places for white matter pathways generated using deterministic streamlines, and therefore do not properly reflect the underlying brain connectivity. This highlights an important limitation of the use of deterministic fibre-tracking algorithms, which can be overcome with probabilistic tracking. 

### 2.5. Image Distortions—Deadly Sin 5

Diffusion-weighted imaging data can often be subject to important image artefacts, including those related to eddy currents and susceptibility-related distortions [52,53,54], as diffusion MRI data are most often acquired using echo-planar imaging. Correcting for these image artefacts during data pre-processing is essential to ensure robust fibre-tracking reconstructions. For example, Irfanoglu et al. [55] demonstrated that using distortion-corrected diffusion MRI data leads to improved accuracy and reproducibility of fibre-tracking results. Similarly, Tournier et al. [22] demonstrated how even apparently small uncorrected errors (e.g., reflected as small deviations on the computed fibre orientations) can constructively propagate to lead to major errors in the tracking outcome (e.g., see example on Figure 10 in reference [22]). 

Correcting for image distortions is also an essential step to be able to combine diffusion MRI with the information from anatomical data (e.g., from high-resolution T1-weighted imaging), such as when using anatomical data to define seed/target regions for fibre-tracking, or to define a gray matter parcellation for connectome analysis, as well as for incorporating anatomical information during tracking, such as with ACT and CMC (see previous section). The image distortions in the echo-planar images need to be corrected for, so that a good spatial match between the diffusion MRI and the anatomical data is possible. 

Importantly, correcting for image distortions in echo-planar imaging does not have to come at the expense of a major increase time in the acquisition protocol (in fact, a correction can be achieved in principle by acquiring just one extra *b*-value = 0 image, with reversed phase-encoding polarity [52]); the inclusion of such extra reference data for distortion correction in diffusion MRI protocols is therefore highly recommended.

Given the major impact it can have on the tractography output, the failure to correct for eddy currents/susceptibility distortions is therefore considered the fifth sin in the list. 

### 2.6. Quantification of Connectivity—Deadly Sin 6

**Figure 6:**
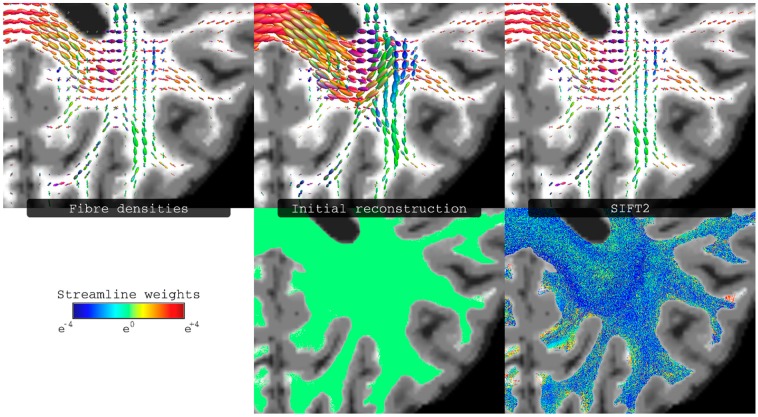
Illustration of the effect of Spherical-deconvolution Informed Filtering of Tractograms 2 (SIFT2) to the reconstructed tractogram. Top row: fibre densities as estimated from either the raw image data (left most), or the streamlines reconstruction (middle and right columns), i.e., representing the streamlines densities also as spherical distributions. Bottom row: streamlines reconstructions, with each streamline coloured according to its weighting coefficient (this is the same value for all streamlines in the initial reconstruction, and the computed streamline-specific weights for the SIFT2 case). Fibre densities shown in top row correspond to the results from spherical deconvolution (left), from the initial streamlines reconstruction (middle), and from the reconstruction with streamline weights determined using SIFT2 (right). Figure modified from a figure previously published in [43], with permission of Elsevier Inc, 2015.

While a simple interpretation of the streamline counts as a quantitative measure of connectivity is very appealing, it has been long recognised that track-count from typical fibre-tracking methods has poor quantitative interpretation, as nicely highlighted by Jones et al. [17]. The main problem relates to the fact that, due to a number sources of error and biases in the tracking process, the local (i.e., at the voxel level) density of streamlines is not consistent with the underlying fibre density [42,56,57] (It should be noted that this inconsistency is independent of what model is used to estimate local fibre orientations, e.g. it affects both DTI-based methods and multi-fibre methods). For example, for the typical acquisition protocols used for high-angular resolution diffusion MRI in clinical systems (i.e., using high b-value and long diffusion gradient pulse durations), the amplitude of the FOD along a given direction can be shown to be proportional to the local intracellular volume [58]. Therefore, at the voxel level, the amplitude of the FOD should ideally match (up to a scaling factor) the streamlines density. Such ideas have been exploited by a number of recent methods [41,42,43,56,59,60,61], to make streamline count a more reliable quantitative parameter. For example, the Spherical-deconvolution Informed Filtering of Tractograms 2 (SIFT2) approach [43] assigns a weight to each streamline in the whole-brain tractogram to globally minimise the mismatch between the FOD and the weighted-sum of streamlines densities (e.g., see Figure 6 for an illustrative example). Using SIFT2, the connectivity between two regions (e.g., the entry in the connectivity matrix of the connectome) can then be computed by summing the resulting weights of the streamlines connecting those two regions. Similarly, this type of approaches can be used for quantifying structural connectivity beyond the analysis of the connectome, such as for the more traditional targeted tracking approach to compute connectivity between user-defined regions (e.g., see Figure 10 in reference [43]).

Importantly, this type of approaches have been shown to reduce the tracking biases [42,43,56,59] and make streamline count a more biological meaningful parameter [62]. 

It is important to emphasise that some alternative (simpler) strategies aimed at addressing the above biases (e.g., the often use of the inverse of the length to compensate for the biases [63]) have been now shown to provide an incomplete compensation for these effects [64], and are therefore no substitute for these more rigorous methods.

Quantifying connectivity by inappropriately counting streamlines is therefore considered the sixth ‘sin’ in the list, and one that must be avoided whenever connectivity quantification is the ultimate aim. 

### 2.7. Binarisation of The Connectome—Deadly Sin 7

The application of fibre-tracking to the study of the connectome has grown considerably in recent years, in what is fast becoming one of the most common applications of fibre-tracking. In this context, given that traditional tracking methods used to be considered unreliable to quantify connectivity (compared with those more advanced methods recommended in the previous section), it became common practice to reduce the information provided by fibre-tracking to a case of absence/presence of connection, i.e., selecting a threshold such that all connections that had streamline count less than the threshold were discarded, and all connections that had streamline count above the threshold were considered present; importantly, all these surviving connections are given the same importance to each other, i.e., the connectome is binarised. To some extent, given the intrinsic quantitative limitations of traditional tracking methods [17], this was a somewhat unavoidable compromise. 

**Figure 7:**
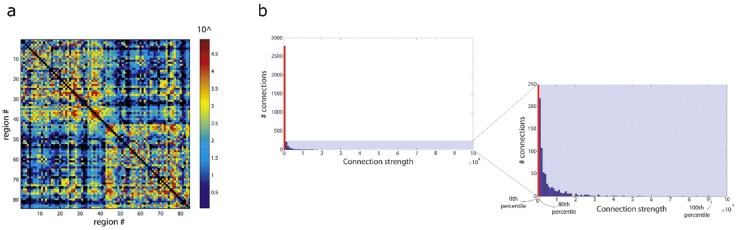
Illustration of the very large dynamic range in connectivity weights in the human brain connectome computed with state of the art fibre-tracking methods. Results obtained from data of the Human Connectome Project using spherical deconvolution, combined with probabilistic fibre-tracking (10 million streamlines computed with the ACT framework), and connectivity quantification using SIFT2, for the case of a 84 node parcellation. (**a**) Connectivity matrix; the colour-scale shows ~5 orders of magnitude in the dynamic range of connection strengths in this example; #: number. (**b**) Histogram of the connection strengths for all edges in the matrix from (**a**): the histogram has a long tail, with strong connections (strengths up to ~90,000) can be several orders of magnitude larger than most connections in the matrix, marked in red (~80% of connections, which have strengths < 1000. Right-most panel shows a zoomed-version of the shaded section of the full histogram. Figure modified from figures previously published in [65], with permission of Elsevier Inc, 2019.

However, with recent advances in tracking methods, which have improved their quantitative properties (e.g., see previous section), the need and/or justification to binarise connectomes has considerably changed. In particular, the original arguments for the binarisation step (namely, fibre-tracking’s poor quantitative qualities) is no longer valid. In fact, recent tracking methods have demonstrated that a huge dynamic range in connectivity strengths are achieved (e.g., see Figure 7), which can expand several order of magnitude between the weaker and stronger connections [65]. The histogram of connectivity strengths has a long tail of very high connectivity strength, and therefore the binarisation step not only requires a somewhat arbitrary decision as to the particular choice of threshold but, importantly, it also discards the wealth of information contained in the computed weighted-connectomes, by setting all connections below the threshold to 0, and all connections above the threshold to 1 (regardless of the latter often having several orders of magnitude difference in their strength). 

Binarising the connectivity matrix in connectome studies is therefore considered the seventh ‘sin’ in the list, as there are currently appropriate fibre-tracking methods to use, such that the full weighted-connectome can now be exploited for subsequent graph-theoretical analysis.

In the context of weighted vs. binary connectomes, there is an important difference between the behaviour of deterministic vs. probabilistic fibre-tracking methods. Zalesky and colleagues concluded that deterministic approaches are beneficial for connectomics, as they provide the best compromise in terms of reducing false positives, at the expense of an increased in the false negative connections [66,67]. While their conclusions were convincing demonstrated, it should be emphasised that they relate to binary connectomes. The situation can be expected to be different for the case of weighted-connectomes, given their intrinsic large dynamic range is likely to reduce the relative contribution from false positive connections (which are most often associated with weaker connections, particularly when using probabilistic fibre-tracking). For example, Civier et al. [65] recently showed that removal of weak connections (often considered as a necessary step to minimise the contribution from false positives) had no statistical effect in commonly used brain network metrics, even after removing ~70–90% of the weakest connections. It is therefore important to have caution when generalising conclusions that have been drawn on binary connectomes to the case of weighted-connectomes. 

## 3. Conclusions

It has been stressed here that, when using diffusion MRI streamlines fibre-tracking for studying brain structural connectivity, there are ‘deadly sins’ that can be avoided at every step of the analysis. While these key issues are often well-recognised in the technical literature, it is not uncommon to still encounter them in application studies. For example, there are sins that can be avoided during acquisition (e.g., deadly sin number 3), others to be avoided during data modelling to estimate local fibre architecture (e.g., deadly sin 1), others during the streamline tracking process itself (e.g., deadly sin 4), others during fibre-tracking quantitation (e.g., deadly sin 6), and finally others more specific to the increasingly common application of connectome analysis (e.g., deadly sin 7). It is recommended that users should be aware of these possible issues with structural connectivity analysis when designing their processing pipelines, and that they are aware of the potential implications their decisions can have on their findings. Avoiding the sins described here should help improve the robustness of structural connectivity analysis using fibre-tracking, as they represent some practical choices a user can do to avoid/minimise these sources of error. 

However, we are not implying here that avoiding the above sins will ensure fibre-tracking is error free. In fact, it should be stressed that, despite major improvements in this field in recent years, even the most advanced fibre-tracking methods currently available still have a number of important limitations, and full validation remains an elusive target [18,68]. Fortunately, fibre-tracking is an area of active research, and there is considerable extra complementary information MRI can provide (both from diffusion MRI data, as well as non-diffusion MRI) to help inform and constrain the complex tracking problem. This has included, for example, recent attempts to incorporate microstructure information [69]. Much is yet to be gained by incorporating further information, both from diffusion MRI data, as well as from non-diffusion MRI (and even non-MRI data), which can help us tackle the very complex problem of robustly characterising brain structural connectivity. We are just scratching the surface; the future of tracking looks bright. 

## Abbreviations

ACT	Anatomically Constrained Tractography
CSD	Constrained Spherical Deconvolution
CMC	Continuous Map Criterion
DTI	Diffusion Tensor Imaging
FOD	Fibre Orientation Distribution
fODF	Fibre Orientation Distribution Function
HARDI	High Angular Resolution Diffusion Imaging
LiFE	Linear Fascicle Evaluation
ODF	Orientation Distribution Function
SIFT2	Spherical-deconvolution Informed Filtering of Tractograms 2
